# Prevalence of dental caries among 6 – 12 year old schoolchildren in social marginated zones of Valencia, Spain

**DOI:** 10.4317/jced.56390

**Published:** 2020-04-01

**Authors:** Ilaria Prada

**Affiliations:** 1 Licensed Dentist at Universidad Europea de Valencia. Master in Pediatric dentistry at Universidad Católica de Valencia, España

## Abstract

**Background:**

To estimate the prevalence of caries and to study oral health habits (oral hygiene, toothbrushing frequency, cariogenic or no cariogenic diet, visits to dentist) in children aged 6 to 12 with social exclusion risk.

**Material and Methods:**

A cross sectional study was conducted in a sample of 160 children aged form 6 to 12 years belonging to Colegio Madre Petra in Torrent and Colegio Nuestra Señora de los Desamparados in Nazaret (Valencia). Among other variables DMFT and Greene and Vermilion simplified plaque index were analyzed.

**Results:**

The prevalence of caries observed was 81.87% and the global DMFT was 4.481. The mean plaque index observed was 1.12. No statistically significant differences were found between global DMFT and sex, global DMFT and age, global DMFT and diet, global DMFT and visits to the dentist and global DMFT and plaque index. A significant association was seen between global DMFT and ethnicity and global DMFT and brushing frequency. Statistically significant association was found also between plaque index and visits to the dentist and plaque index and diet.

**Conclusions:**

It was observed that children at risk of social exclusion had a very high global DMFT and a regular plaque index. So, it can be concluded that social exclusion constitute an underlying factor that increase caries prevalence and global DMFT and a marker of poor oral hygiene habits.

** Key words:**Dental caries, caries prevalence, oral hygiene habits, plaque index, toothbrushing frequency, social exclusion.

## Introduction

Dental caries is a polymicrobial dysbiotic pathology characterized by a chronic-degenerative process originated by multifactorial etiology (1). Furthermore, it is one of the most widespread diseases in the global population (2) and it’s the most frequent chronic pathology in childhood (3). It is characterized by a local destruction of the dental hard tissues produced by acidic products derived from the bacterial fermentation of carbohydrates assimilated from diet (2). This destruction is originated from a dynamic process in which demineralization and remineralization periods rapidly alternate (3).

To produce a carious lesion, one or various of these etiological factors defined by Keyes in 1963 should coexist: cariogenic bacteria, susceptible host and substrate, that is fermentable carbohydrates. However, Fejerskov and Manji in 1990 observed that the biological factors weren’t the only causes of the carious pathology, and that’s why behavioral and socioeconomic factors may also cause the disease (4).

On the other hand, social exclusion is defined by the Plan Nacional de Acción para la inclusión social del Reino de España (PNAin) de 2013-2016, as “a process of loss of people integration or participation in society and in different political, economic and social spheres”. The PNA claims that poverty is a variable that is capable of affecting, to a lesser or greater extent, social exclusion; however, not all poor people are socially excluded and vice versa (5). There are several risk factors that can increase the possibility of being part of the social exclusion process and they are: low income, lack of family networks, problems at school, residing in a disadvantaged neighborhood, suffering from mental illness, being elderly, having mental or physical disability and being part of a vulnerable group (6).

Individuals included in social exclusion groups may have less education, lower income level, poor access to services, stress, poor health (inadequate physical health due to poor diet, lack of physical exercise, abuse of tobacco and drugs), lack of hope and reduced social cohesion (6). All this, consequently, influences the oral health, in particular on the possibility of accessing preventive measures such as toothbrushes, dental floss, a diet with low intake of sugars and calories, the application of fissure sealants and of fluorine (7).

Children with social exclusion risk may present factors that make them more susceptible to certain oral diseases, such as caries, and poor oral hygiene. These factors are mainly associated with the environment that surrounds them, that is, the level of education and beliefs of parents, family income, domestic problems, situations of abuse, the absence of parents, etc. All this, on the one hand, influences the possibility of accessing preventive and health measures, and on the other hand, the lack of interest that parents experience for the oral health of their children. Several studies have already shown the association between a low socioeconomic level and a higher DMFT/dmft (8-10).

Therefore, it would be interesting to find out if the same situation occurs in Spain, particularly in the Valencian Community. In addition, this study also aims to relate the concept of social exclusion with the level of oral hygiene and diet carried out by children belonging to these communities.

Thus, the present work arises as a response to the need to check whether there is a higher prevalence of caries, a deficit in oral hygiene habits and an unbalanced diet in children with social exclusion risk. It has also been developed to increase sensitivity to and awareness of both the preventive and treatment needs which are present in this population group.

In particular, the aims of this study are: to estimate the prevalence of caries and to study oral health habits in children aged 6 to 12 years with social exclusion risk.

## Material and Methods

-Study type

This was an observational and cross sectional study, also known as prevalence survey.

Sample size and selection

The study population was primary school children aged 6 to 12 years belonging to two different schools in Valencia: Madre Petra School managed by the association of gypsies and marginalized children of Torrent and Nuestra Señora de los Desamparados in Nazaret.

Since the population of this study is an unknown population, the estimated sample size is 150 children, an estimation obtained by establishing the 95% confidence interval, the expected frequency at 0.5, and the expected error of 0.07.

After carrying out the field work and cleaning data, the resulting valid sample contained 160 pupils. The population was balanced between sexes (males 52.5% and females 47.5%; Z-test *p*-value 0.37) and the average age was 8.96 years.

-Data collection 

The examinations were performed in the aforementioned schools, in the spaces assigned for this purpose. The examinations took place with the child sitting on a chair, with his or her neck extended, and the examiner placed behind or in front of him/her. While the examiner conducted the examination by speaking the results aloud, the recorder sat alongside and filled in the examination record. Illumination was constant during all the examination sessions, as the teams were provided with a portable head lamp. The examination instruments employed were a WHO-type periodontal probe and a no. 5 plain mouth mirror. The examining team was provided with 40 sterilized probe and mirror sets, each in a sealed bag. The field work was carried out between November 2018 and March 2019.

-Ethical aspects

Before beginning the field work, a project program was written and presented to the university ethical committee, who accepted the investigation with the number UCV/2018-2019/011. In the meantime, presentation letters, informed consents and informed assent were prepared and given to the school directors some time before data collection. The two first documents were directed towards parents to explain what the examination consisted in and to obtain their consent to make their child participate in the study. On the other hand, informed assent was given to 12 years old children to obtain their permission to check their mouth. However, full availability to give further information was offered, almost half of the potential sample was lost due to the lack of consent form return. In fact, children, even though they had been notified in advance, didn’t bring back the consent form by the exploration day deadline because their parents didn’t sign it due to lack of interest or maybe comprehension. It was also observed that some consent forms were stained or were written and signed in capital letters (illiteracy indication).

-Study variables

To determine the presence of caries on the dental surfaces, the criteria established by the WHO were followed, considering, therefore, a decayed tooth when it showed a shadow under the enamel, or a cavitated lesion or undermined enamel; the incipient lesions of white spot were not considered caries. The dmft and DMFT indexes were used to evaluate the affectation of the temporary and permanent dentition respectively; since the majority of the population had mixed dentition, it was considered more representative to use, to compare with other variables, the summation of dmft + DMFT in temporary and permanent dentition.

To assess oral hygiene, the Greene and Vermillion Simplified Index (IHO-S) was applied, in which the coronary extension of the plaque was estimated.

In order to record the diet consumed by the children, the record called 24-hour reminder has been used, in which the children have to remember what they ate and drank the previous day. In order to be able to carry out the statistical analysis successively, those diets that had three or less sugar intakes was considered non cariogenic while the ones that presented more than three intakes were considered cariogenic.

Furthermore, age, sex and gender data were collected. The ethnicity was classified in different categories based on the parents’ origins. Finally, children were asked about tooth-brushing frequency and if they had ever gone to the dentist.

-Statistical analysis

The data obtained were analyzed using the SPSS 23.0® statistics package to compute descriptive statistics with means and proportions, with 95% confidence intervals for each. For the inferential statistics, T-Student or ANOVA tests were used to compare means and the chi square test was used to compare proportions.

## Results

**Figure 1:**
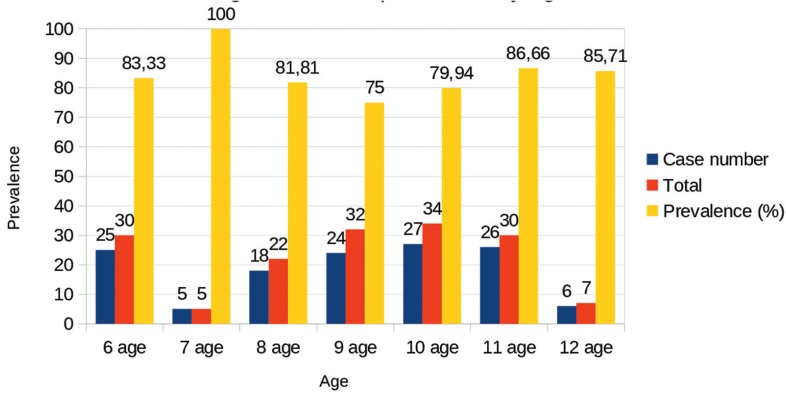
Caries prevalence by age.

**Figure 2:**
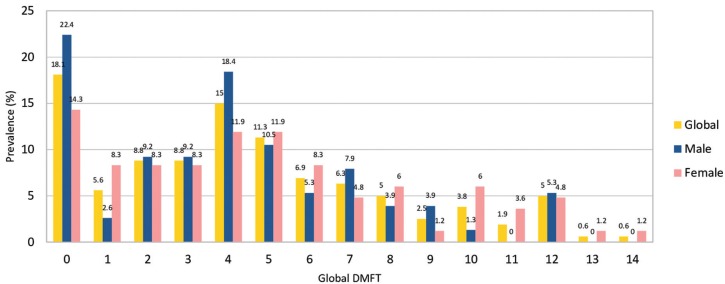
Global DMFT as a categorical.

The caries prevalence in total dentition was 81.87% while caries prevalence by age is shown in Figure 1. The global DMFT score (both primary and permanent) was 4.48, whereas the results obtained considering it a categorical variable are shown in Figure 2. No statistically significant differences were shown by sex and age; nevertheless, at 9 and 10 years children presented less caries and the global DMFT was inferior in males. Decayed teeth were the majority component for both DMFT and dmft, with a 72.39% and a 74.02% respectively, while filled teeth corresponded to 26.75% in DMFT index and to 18.43% in dmft.

The predominant ethnicity was the Spanish one (33.1%) followed by Gypsy (Romani) (27.5%) and Latin American (20.6%). A statistically significant association was found between the global DMFT and ethnicity (Chi Square test; *p* <0.05), being, among the three most prevalent ethnic groups, Spanish children, followed by Latin American, who presented less tooth decay.

On the other hand, the mean IHO-S was 1.12 (0.1 – 2.1) but no statistically significant differences were found with age, sex and ethnicity and neither with global DMFT.

Analyzing oral health behavior we found that 44.38% of children never brush their teeth and only 11.25% of them did it three times per day. Instead 68.13% of children told that they had ever gone to the dentist, but asking them how many times they had gone, the majority said that they had gone only one time. Finally 84.38% of the population was considered to carry out a non cariogenic diet (less than 3 sugars intakes per day).

Tooth-brushing habits were found to be statistically significant associated with global DMFT: children that brushed their teeth 3 times per day had a global DMFT 2.93 units lower (95% CI 0.55-5.32) than those who didn’t brush. However, dentist visits and diet were not associated with global DMFT, which means that there weren’t statistically significant differences between children that had gone to the dentist ant the ones that had never gone and between cariogenic and non cariogenic diet regarding global DMFT (Table 1).

**Table 1:**
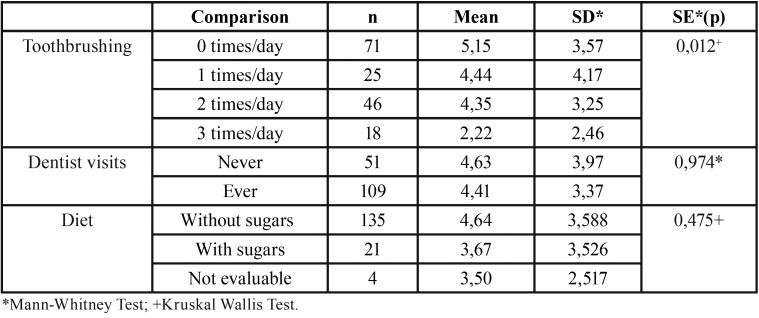
Global DMFT analysis in relation to toothbrushing, dentist visits and diet.

On the other hand, we have the results obtained comparing the three oral health habits variables and IHO-S index. No statistically significant differences were found in the plaque index with respect to the tooth-brushing variable; but there was significance in going to the dentist and the diet.

The plaque index of those who had gone to the dentist was 0.189 times lower (95% CI 0.048-0.33) than those who have never gone. The same occurs for the diet variable, but in this case, it was found that the plaque index of those who had a diet with sugars was 0.250 times lower (95% CI 0.06-0.44) than those who did not consume sugars (Table 2).

**Table 2:**
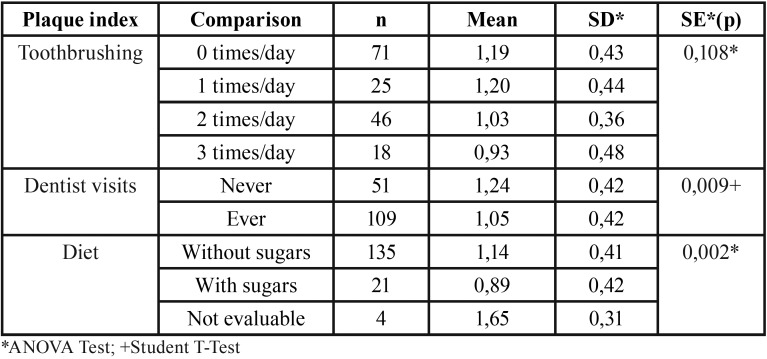
Plaque index analysis in relation to toothbrushing, dentist visits and diet.

No statistically significant differences were found in tooth-brushing frequency with respect to sex, but there were significant differences with respect to age (at an older age, they brushed more) and with respect to ethnicity (Spanish and Latin American children brushed more than the rest). Neither were there any statistically significant differences in tooth-brushing found depending on the diet type the children carried out. On the other hand, there was a statistically significant association between the tooth-brushing frequency and having gone to the dentist: those children who had gone to the dentist brushed their teeth statistically more times compared to those who had never done so (Table 3).

No statistically significant differences were found in having visited the dentist with respect to sex, ethnicity and diet, but statistically significant differences were found with respect to age (the older, the more they visited) (Table 4).

Neither did we find statistically significant differences in diet with respect to sex or age, but we did find it with respect to ethnicity (the majority of ethnic groups use sugar-free diets).

**Table 3:**
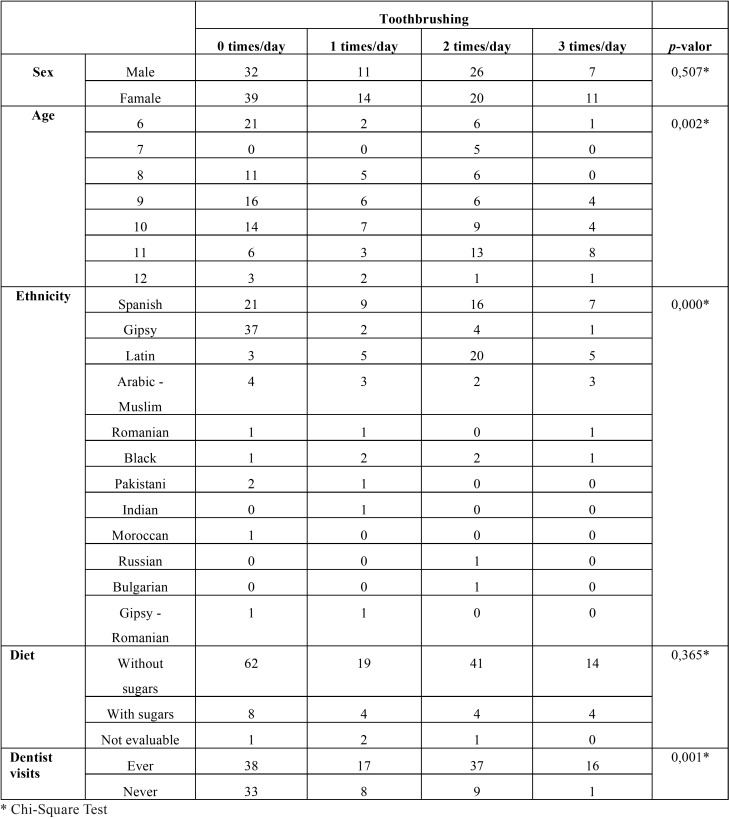
Toothbrushing analysis in relation to sex, age, ethinicity, diet and dentist visits.

**Table 4:**
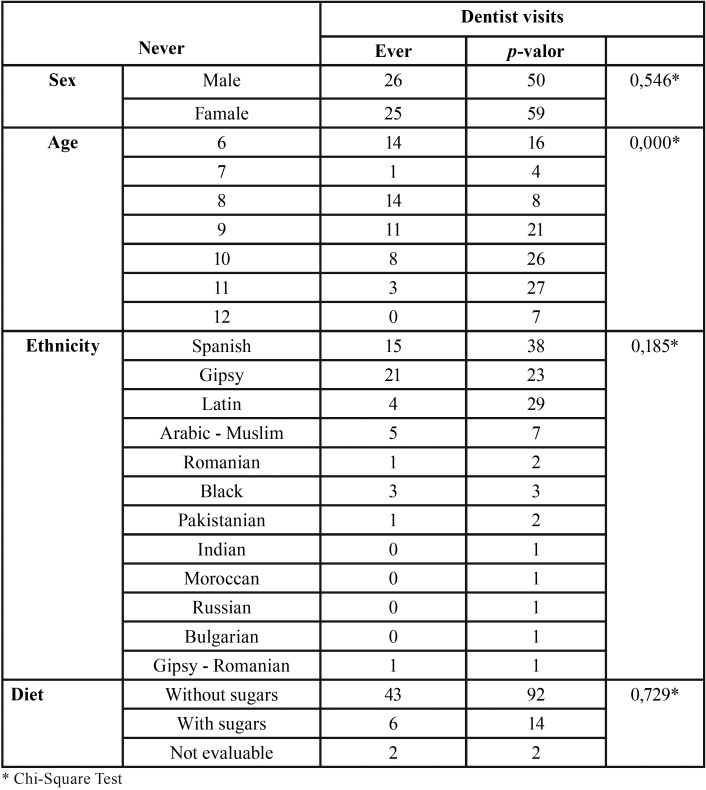
Dentist visits analysis in relation to sex, age, ethinicity and diet.

## Discussion

The two schools chosen for the study accept children belonging to groups at social exclusion risk, such as gypsies, people with limited financial resources, families with problems of addiction and imprisonment of one or both parents etc.; therefore, the two institutions try to favor the process of education and social integration of the children with facilities, such as the providing of breakfast, meals and snacks, and the distribution of clothing and school reinforcement programs.

In the present study, it has been decided to study dmft and DMFT as qualitative and non-quantitative variables because the sample analyzed was small, and it was considered more representative to describe the distribution of the variable rather than its mean, since, in this way, it gave much more information.

In addition, it was decided to combine the dmft with the DMFT since the majority of the population analyzed had mixed dentition and therefore it was more interesting to analyze the index of caries, fillings and missed teeth per person, rather than per dentition.

The caries prevalence of the two studied groups is 81.87%. This result is comparable to that obtained by Ballouk and Dashash (11) in 2019 (n = 1500), which found a caries prevalence in Damascus (Syria) of 79.1%, 91.14% of the DMFT and 89.1% of the dmft were attributed to the decay and missing teeth values, while in the present study the corresponding values are 73.24% and 81.55%.

In the s2,tudy by Kumar *et al.* (12) the caries prevalence in children aged 6 to 12 in the district of Udaipur (Rajasthan) was a little lower, 64.9%, being, as in our study, the decay tooth component was the dominant one in the global DMFT index.

Also in the study by Almerich and Montiel (13), (n = 1388), in 2006, the missing tooth component was the dominant one, being 82.5% in 6-year-olds, 84.2% in those aged 12 and 90.9% in those aged 15-16 in temporary dentition and 73.5% at 6 years, 65.4% at 12 years and 65.4% at 15-16 years in permanent dentition. In our study, on the other hand, component d and D was not divided by ages, but was calculated globally and resulted in 74.02% in temporary dentition and 72.39% in definitive dentition.

The prevalence of caries is lower in the study by Sakeenabi *et al.* in 2012 (8), 28%, and these authors, as well as Sudha *et al.* (14), in 2005, found no statistically significant association between sex and caries prevalence. On the other hand, the study by Kumar *et al.* (12) also saw that there was a statistically significant association between caries prevalence and sex, being, the male sex that had a higher global DMFT compared to the female sex. The study by Gatou *et al.* (15), in 2011, on the other hand, obtained opposite results, with the female sex having the highest caries experience.

David *et al.* (16) study which revealed a caries prevalence in Thiruvananthapuram (India) of 27%, found no statistically significant differences between sexes with respect to caries prevalence.

Almerich and Montiel (13) neither found a statistically significant association between caries experience and sex. However, the prevalence of caries was much lower compared to our study: 34.2% at 6 years, 48.3% at 12 years and 56.1% at 15-16 years, compared with 83.33 % in children of 6 years and with 85.71% in children of 12 years in our study. This data shows that there is a great difference between the caries prevalence between children in Valencia city and children belonging to groups at risk of social exclusion within the same society.

Numerous studies (15,17), agreeing with our results, proved that children belonging to foreign families and ethnic minority groups had a higher prevalence of caries due to lack of knowledge in the oral health field, different beliefs and a difficult access to care services.

Chi *et al.* (18) demonstrated that children who lived in environments that could not afford to have adequate food security for their needs had a higher prevalence of untreated caries compared to others. This can be justified through four possible explanations. First, food insecurity pushes families to choose quantity and not quality, for example, a juice or a soda is accessible and cheap but not healthy as long as they contain a large amount of sugar. Second, the neighborhoods where these families live usually have few supermarkets and points of sale of fresh food, but rather mini-markets with packaged food, junk food etc, which deprives children of having access to fruit, vegeTables, fish and meat, forcing them to opt for things such as chocolate bars, cookies, sweets, etc. Third, children who experience food insecurity tend to have smaller but more frequent meals so that food can last longer (the increased intake frequency exposes the teeth to a more repetitive acid attack). Fourth, having food insecurity is associated with less social capital and greater biological stress, which also intervene in an increase in caries prevalence.

This is the reality in which the children of Colegio Madre Petra and Nuestra Señora de los Desamparados live. Fortunately, the two schools serve at least two meals a day (breakfast and lunch or lunch and snack), so during the week, the possibility of exceeding the three sugar intakes considered cariogenic is controlled and reduced. This is the reason why the diet of most children, 84.38%, has been considered sugar free. However, despite this, the calculated caries prevalence was very high and there was no statistically significant differences (*p*> 0.05 Kruskall Wallis test) between the cariogenic and non-cariogenic diet groups regarding global DMFT. This result is probably due to the fact that the data were collected with a 24-hour reminder done intra-weekly and asked only to the children. It would have been more reliable to carry out this evaluation with a dietary diary since in this way the real diet of the children would have been collected for a week, including weekends, when the diet was not regulated by the schools, and it would also have been convenient to involve parents. However, given the characteristics of the analyzed group (very little parental cooperation, negligence, lack of education) it was not possible to give out this type of dietary survey.

Another factor that may explain a high caries prevalence, as suggested in the study by Subramaniam and Singh (19) (n = 2033), is that children belonging to families with a low SES, as in the case of the children studied in our research, have a very low intake of cario-protective foods such as fruits, vegetables, milk and dairy products. What is more, probably, during their childhood and training period they may have had an unbalanced diet, with a lack of vitamins A and D, calcium and phosphate, which contributes to the alteration of the balance of the oral ecosystem, increasing the caries risk, especially in temporary dentition.

A study that, unlike our results, found a statistically significant association between global DMFT and diet is that of Creske *et al.* (9) (n = 177); however, their data was based on a questionnaire filled out by the mothers in which they asked, for example, the number of snacks consumed per day, the amount of complex carbohydrates ingested, the type of drink consumed, etc. What is comparable to our results is that they did not find a statistically significant association between diet and sex, diet and brushing and diet and visits to the dentist.

The study of Sakeenabi (8) confirmed that there is a statistically significant association between a diet with sugar consumption and an increase in caries prevalence. However, these authors collected the children’s diet in a 24-hour reminder given out to the parents and considered a diet with sugar when the children consumed one or more foods that contained sugar such as snacks or juices.

Also in the study by David *et al.* (16) a statistically significant association was found between caries prevalence and diet, being the highest in those children who consumed sugars more frequently.

Punitha *et al.* (20), in 2015, used a consumption frequency questionnaire, integrated with other questions, to determine an association between diet and DMFT in 12-year-old children. They found that the foods that were statistically most associated with a higher DMFT were soft drinks and pastries that included chocolates and sweets; instead, they found no association with intake of sugar, milk, fruits and vegetables, non-vegetarian foods, junk food and freshly squeezed fruit juices.

Quadri *et al.* (21) (n = 500), saw that not only the level of sugar ingested per day marked statistically significant differences in the DMFT, but also influenced the frequency of meals per day and the amount of snacks between meals; DMFT was higher in those children who had a high sugar intake, who ate 1 or 2 times a day and who consumed more than 1 snack.

Also the study by Sudha *et al.* (14) confirmed that both dmft and DMFT increased as the number of sugar exposures increased, being higher in those children who ate sugar-rich foods 4 or 5 times a day. In addition these same authors saw statistically significant differences between dmft and DMFT, the influence of sugar was much more significant in the DT than in the DP.

Giving out a 24-hour reminder and considering both the consistency of food (soft/liquid, sticky or solid) and the amount of sugars contained, Gupta *et al.* (22), in 2014, did not find a statistically significant association between diet and global DMFT.

Sex does not seem to be a clear indicator of children’s food preferences and their inclination to choose foods with more or less sugar. It must be considered that, especially children of younger ages tend to eat what parents offer; therefore, their diet is influenced by their family’s choices.

Furthermore, diet can be influenced by ethnicity and by the culture of the origin country that may lead to preferring some foods over others; however, the influence that the country of residence exerts is quite relevant, given that the different immigrant populations usually incorporate in their diet typical foods of the country that welcomes them and they adopt their eating habits.

A study that highlights the poor oral hygiene of the children analyzed in our study is that of Do *et al.* (10) since, in their study, it was found that 73.4% of children brushed their teeth more than twice a day and that only 26.9% brushed less than twice a day. However, Do *et al.* found a statistically significant difference in the prevalence of tooth decay between children who brushed their teeth more than twice a day and those who did less than twice, the former having the highest index. These results are comparable with those obtained in our study since it was found that children who never brushed their teeth had a global DMFT 2.93 times higher than those who brushed 3 times a day.

Also in the study by Sakeenabi *et al.* (8) a statistically significant association was found between brushing and caries prevalence, being higher in those children who brushed their teeth less than 2 times / day compared to those who brushed 2 or more times a day.

Therefore, most authors determine that there is an association between the frequency of brushing reported by children and the prevalence of caries, this relationship being inversely proportional.

Regarding the association between brushing frequency and sex, it seems that in most studies, as claims Chu *et al.* (23), differing form our results, girls are who tend to brush their teeth more frequently, which is probably due to a greater concern for their oral health and aesthetic appearance.

Authors like Petersen *et al.* (24) also confirm this, as in our study, the association between brushing frequency and ethnicity, mainly due to cultural beliefs, to socioeconomic level (SEL) and to the parents’ knowledge.

However, researchers did not find unanimity regarding the association between brushing frequency and age, in many studies, like in that of Pujar *et al.* (25) it has been seen that an older age is associated with more effective brushing with better plaque removal.

The rate of those who have never gone to the dentist, 31.87% in our study, is higher than that found by Creske *et al.* (9), which is 1.1%; the remaining 98.9% had ever attended the dental clinic, however 24% of the parents said that after the first visit they could not cope with the necessary cures for their children’s oral health. Their results, like ours, revealed that there was no statistically significant association between visits to the dentist and the global DMFT.

John *et al.* (26) also did not find a statistically significant association between visits to the dentist and DMFT, but they found it between having visited the dentist and dmft, being the highest caries index in those children who had never attended a clinic. They also saw that the majority of children analyzed, 62% of children in tribal schools, 68.9% and 60.6% of children in suburban and urban schools respectively, had never gone to a dentist. The reasons why they had never visited a dental clinic concur with what we observed, can be summed up in the following: fear, lack of parents’ dental knowledge, family income, lack of infrastructure, false beliefs in merit of dental treatments due to illiteracy. On the other hand, the study by David *et al.* (16), unlike ours, found a statistically significant association between the prevalence of tooth decay and having visited a dentist, being higher in those children who had attended a dental clinic, 33%, compared to those who had never done so, 23%.

In particular, it seems that younger children and ethnic minorities are the ones who visit a dentist less frequently (27); the reasons may be, among others, the belief that baby teeth do not have to be cured since they fall, lack of access to services and social exclusion experienced by minor ethnic groups.

The mean plaque index found in our study is 1.12, which is considered regular, according to the simplified Greene and Vermillon index. However, there was no statistically significant association between this and the global DMFT (*p* = 0.059). These results are probably due to the fact that the IHO-S analyzed the plaque present only on the free surfaces, while caries originate meanly in the occlusal and interproximal surfaces. Nevertheless, it is true that many children who had a very high plaque index did not have cavities while several children who had a high number of cavities had a very low plaque index. The study of David *et al.* (16), as well as that of Gupta *et al.* (22) corroborate our results, not finding a statistically significant association between plaque index and caries prevalence. On the other hand, Sudha *et al.* (14) saw that the dmft increased with the increase of the plaque index until it reached a value of 1.9; while, when the plaque index was between 2 and 3, the dmft decreased; DMFT also increased with increasing plaque index.

In our study, no statistically significant association was found between plaque index, age, sex, ethnicity, etc. However, it was found between the plaque index and having gone to the dentist, the plaque index is 0.189 lower in children than had gone to the dentist, and the plaque index and diet, being the first 0.25 times lower in children who consume diets without sugars.

Analyzing different studies, plaque index seems to be higher in the male sex (15,21) and in the children with lower age (15), probably due to a lower concern and a lower manual ability.

Crossing the variables of tooth brushing habits and plaque index, in our study, as well as that of Gopinath *et al.* (28), no statistically significant association has been seen.

Quadri *et al.* (21), unlike our study, found a statistically significant association between the two variables, being plaque index higher in those children who brushed less than once a day. When evaluating this relationship, more than the frequency of brushing, the effectiveness of brushing when removing the plaque appears to be significant.

In our study it was seen that plaque index was lower in those children who had said that they had gone to the dentist at least once. This may probably be due to the fact that children who have gone to a dental clinic may have received oral hygiene instructions.

The main limitation of the present study was the fact that all data were obtained by interviewing children due to the lack of parental collaboration. Therefore the information obtained regarding, above all, sugar intake and brushing frequency may not be entirely reliable. So it would be necessary to carry out a study with a larger sample number and giving out the questionnaires to parents. It would also be very interesting to conduct a comparative study with children without social exclusion risk.

## Conclusions

The caries prevalence of the population studied is 81.87% with a community global DMFT of 4.48. There was a statistically significant association between caries prevalence and ethnicity (children with less caries were Latin American and Spanish) and brushing frequency (children who brushed 3 times/day presented 2.93 less caries compared to those who never did). No association was found between caries and diet and plaque index.

The average plaque index was 1.12. There was a statistically significant association between this and the diet and having gone to the dentist (the plaque index was lower in those children who had visited a dental clinic compared to those who had never gone).

The oral hygiene habits of the children studied were negligible; in fact, 44.38% of them said they never brushed their teeth. However, it was seen that brushing was statistically associated with age, the older the higher the frequency of brushing, with ethnicity, Spanish and Latin American children were the ones who brushed the most, and having gone to the dentist, the children who had gone to a dental clinic brushed more frequently.

Therefore it can be concluded that social exclusion constitutes an underlying factor that increase caries prevalence and global DMFT and is a marker of poor oral hygiene habits.
